# Shallow or deep? The impact of orthographic depth on visual processing impairments in developmental dyslexia

**DOI:** 10.1007/s11881-021-00249-7

**Published:** 2022-03-14

**Authors:** Serena Provazza, Barbara Carretti, David Giofrè, Anne-Marie Adams, Lorena Montesano, Daniel Roberts

**Affiliations:** 1 School of Psychology, Liverpool John Moores University, Liverpool, UK; 2 Department of General Psychology, University of Padova, Padua, Italy; 3 Department of Educational Sciences, University of Genoa, Genoa, Italy; 4 Università Della Calabria, Rende, Italy; 5 Centre for Cognitive Neuroscience, Division of Psychology, College of Health, Medicine and Life Sciences, Brunel University London, Uxbridge, UK

**Keywords:** Developmental dyslexia, Orthographic depth, Phonology, Visual processing, Visual word form area (VWFA)

## Abstract

The extent to which impaired visual and phonological mechanisms may contribute to the manifestation of developmental dyslexia across orthographies of varying depth has yet to be fully established. By adopting a cross-linguistic approach, the current study aimed to explore the nature of visual and phonological processing in developmental dyslexic readers of shallow (Italian) and deep (English) orthographies, and specifically the characterisation of visual processing deficits in relation to orthographic depth. To achieve this aim, we administered a battery of non-reading visual and phonological tasks. Developmental dyslexics performed worse than typically developing readers on all visual and phonological tasks. Critically, readers of the shallow orthography were disproportionately impaired on visual processing tasks. Our results suggest that the impaired reading and associated deficits observed in developmental dyslexia are anchored by dual impairments to visual and phonological mechanisms that underpin reading, with the magnitude of the visual deficit varying according to orthographic depth.

## Introduction

Developmental dyslexia (DD) is a neurodevelopmental disorder and the most common specific learning disability, affecting ~ 15% of people globally (American Psychiatric Association., 2013). It is characterised by an unexpected inability to achieve fluent, accurate reading (Lyon, 2003; Ramus, 2003b). Although most of the research regarding DD has been conducted with children, reading difficulties persist into adulthood (Bruck, 1985; Eloranta 2018; Finucci et al., 1985; Nergård-Nilssen & Hulme, 2014; Shrewsbury, 2016).

The commonly accepted explanation for reading impairments in DD is a deficit in phonological processing (see Elliot & Grigorenko, 2014, for a review; Snowling, 1981, 1995; Stanovich, 1988; Stanovich & Siegel, 1994; Swan & Goswami, 1997; Vellutino et al., 2004). In developmental terms, this deficit is considered to affect the acquisition of phonological decoding skills (i.e. grapheme to phoneme conversion), which prevents construction of the orthographic lexicon, thus impacting fluent, whole-word recognition. A phonological deficit typically encompasses impaired phonological awareness, verbal short-term/working memory, and/or letter knowledge, three of the most widely studied measures of phonological skills in developmental reading disorders (Hulme & Snowling, 2013; Melby-Lervåg et al., 2012; Snowling & Melby-Lervåg, 2016).

Verbal short-term memory supports the time-limited storage of verbal information (Snowling & Hulme, 1994; Unsworth & Engle, 2007), whereas verbal working memory involves the manipulation of this information (Baddeley, 1986; Baddeley & Hitch, 2000). With respect to reading, efficient operation of phonological codes in memory is necessary for segmenting and blending sounds in spoken words, especially important when learning to read (see Baddeley, 1986; Gathercole & Baddeley, 1993; Melby-Lervåg et al., 2012). As such, verbal short-term memory and working memory play a crucial role in reading and if impaired may lead to reading difficulties (Macaruso et al., 1996; Trecy et al., 2013).

Accumulating evidence on the anatomical basis of acquired and developmental reading disorders has confirmed a variety of regions are involved, predominantly in the parieto-temporal area (such as the angular gyrus, supramarginal gyrus, and posterior portion of the superior temporal gyrus, Damasio & Damasio, 1983; Paulesu et al., 2001). These areas are associated with phonological processing, which implies that there may be a universal neurocognitive basis for DD, and suggests a core phonological deficit among individuals with DD, irrespective of orthographic depth (Carroll & Snowling, 2004; e.g. Goswami, 2002; Paulesu et al., 2001; Snowling & Hulme, 1989; Vellutino, 2004).

Although phonological deficits appear to represent a significant component of developmental reading difficulties (Paulesu, 2001; Ziegler et al., 2003), some cross-orthographic differences have been noted. Wimmer (1993), for instance, distinguished between (1) speed dyslexia, in which the prevailing phonological deficit manifests in dysfluent and slow word recognition with accuracy being affected to a lesser extent, characterising DD in shallow orthographies (i.e. orthographies with consistent spelling-to sound mapping, e.g. Italian), and (2) decoding dyslexia, in which the phonological deficit results in incorrect word decoding, which characterises DD in deep orthographies (i.e. orthographies with an inconsistent spelling-to-sound mapping, e.g. English).

Despite the well-established role of impaired phonological processing in DD, the contention that a phonological deficit per se may account for the impairments in DD is disputed (Bosse, 2007; Elliot & Grigorenko, 2014; Giofrè et al., 2019b; Provazza, 2019c; Valdois et al., 2004). Some authors suggest that phonological skills are intact but become inaccessible depending on the task requirements. A review conducted by Ramus and Szenkovits (2008) found that individuals with DD performed poorly on short-term memory tasks, tasks requiring the conscious manipulation of the phonological representation, or those conducted under time constraints. Thus, whilst not questioning the traditional interpretation of the core phonological deficit in DD, the authors emphasise that the phonological deficit has an access quality, with the phonological system remaining relatively intact despite an impairment in the processes by which the system is accessed or activated.

Although phonology appears to be a contributing factor in DD, some studies demonstrate that a phonological deficit may be less important (Elliot & Grigorenko, 2014; Giofrè et al., 2019b; Provazza et al. 2019b; Smith-Spark et al., 2003; Vidyasagar & Pammer, 2010). For instance, not all individuals with reading disabilities demonstrate a phonological deficit (Castles & Coltheart, 1993; Frederickson & Frith, 1998; Frith, 2017; White et al., 2006; Wolf & Bowers, 1999) and individuals with poor phonological abilities can nevertheless become competent readers (Elliot & Grigorenko, 2014; Howard, 1996). Accordingly, reading acquisition may involve a variety of factors extending beyond linguistic components (e.g. Verhoeven et al., 2011) highlighting the salience of a multi-faceted account of the deficit as well as for other developmental disorders (see Astle & Fletcher-Watson, 2020; Elliot & Grigorenko, 2014; Gibbs & Elliott, 2020). Specifically, DD may also be characterised by other underlying impairments, such as a visual processing deficit that would prevent fluent word reading (Provazza et al., 2019b; Sigurdardottir et al., 2018; Stein & Fowler, 1981; Valdois et al., 2003).

There has been a longstanding interest in the role of visual processing in DD (Hinshelwood, 1896; Lovegrove, 1993; Martin & Lovegrove, 1984; Orton, 1925), and a number of different forms of visual processing deficit have been suggested. Reading requires fast and accurate visual processing, and some researchers have proposed that DD might be associated with a deficit in the magnocellular visual system (Livingstone et al., 1991; Stein, 2018; Stein & Fowler, 1981; Stuart et al., 2012). Indeed, individuals with DD are impaired in tasks using rapidly presented non-orthographic visual stimuli (Livingstone et al., 1991; Stein et al., 1999). Consequently, a deficit to the magnocellular pathway may account for poor performance on a range of visual tasks, and evidence supports the association between reading difficulties in DD and a dysfunctional magnocellular system (see Elliot & Grigorenko, 2014, for a review). Although the magnocellular deficit hypothesis received significant attention, it has remained controversial (Ramus et al., 2003; Stuart et al., 2012). For instance, many individuals with magnocellular deficits were able to develop adequate reading skills (see, e.g., Skoyles & Skottun, 2004). Furthermore, a magnocellular deficit has been reported in the phonological dyslexia subtype which is characterised by impairments when reading unfamiliar words and non-words but fails to account for the impairment in the surface dyslexia subtype, a selective difficulty reading irregular words (Bosse et al., 2007; Facoetti et al., 2006; Ramus, 2003a; Valdois et al., 2003).

An alternative account, the visual attention span hypothesis, provides a more encompassing explanation of the reading impairments in DD and its subtypes (Bosse et al., 2007). The visual attention span is conceived as the amount of visual information that can be processed in parallel (Bosse et al., 2007), with impairments of visual span accounting for reading disorders independently of a phonological processing deficit (Bosse et al., 2007; Valdois et al., 2003; Vidyasagar & Pammer, 2010). Interestingly, a deficit in visual attention span has been shown to affect not only the recognition of verbal letter strings but also other kinds of stimuli, such as numbers and non-verbal symbols (Lobier et al., 2012; Vidyasagar & Pammer, 1999). This suggests that a visuo-attention span deficit is a consequence of impaired visual processing rather than an orthography-to-phonology mapping deficit.

However, the evidence of impaired visual attention span in DD has been questioned (Banfi et al., 2018; Ziegler et al., 2010). The principle issue concerns the methodology employed (Lobier et al., 2012; Ziegler et al., 2010). Visual attention span tasks often rely on verbal material (letters or digits). Impairments on this material, but not on non-verbal symbols (Ziegler et al., 2010), might be explained by the visual-to-phonology mapping employed in these tasks (although it should be noted that such a link has not always been confirmed; see Banfi et al., 2018; Collis et al., 2013). Such effects cannot be considered to arise unambiguously from a visual processing deficit. Moving forward, it is therefore important to assess DD readers on tasks sensitive to identifying visual processing impairments and which are unambiguously not contaminated by visual-phonological processing.

Such evidence of a specifically visual processing deficit in DD has been documented by studies investigating non-reading visual impairments that may be attributed to a dysfunctional left occipito-temporal cortex. This dysfunction not only reduces rapid and automatised recognition of letters and words (similar to the proposal of Wimmer, 1993) but is also implicated in speed of processing for other demanding visual stimuli (Behrmann & Plaut, 2013, 2020; Behrmann et al., 1998; Kronbichler et al., 2008; Price & Devlin, 2011b; Sigurdardottir et al., 2021). For instance, this region is responsive when processing non-orthographic visual stimuli, such as faces and visually complex objects (Behrmann & Plaut, 2013, 2020; Gabay et al., 2017a; Roberts et al., 2013, 2015)—stimuli which DD individuals are slow to process. Meta-analytic studies in shallow and deep orthographies consistently identified hypoactivation of this region (Martin et al., 2016; Richlan, 2014) which may result in both reading and non-reading deficits if tested appropriately (Giofrèet al., 2019a; Jozranjbar et al., 2020; Sigurdardottir et al., 2018; Vogel et al., 2014).

Of particular interest within the occipito-temporal cortex is the visual word form area (VWFA), and it is well-established that fluent reading relies heavily upon this region (Centanni et al., 2019; Cohen & Dehaene, 2004; Dehaene & Cohen, 2011; Schurz et al., 2010). The VWFA operates on the whole-word (word form) recognition level (Pugh, 2006; Pugh et al., 2000), although some research found it is also activated during sub-lexical processing (Martin et al., 2016; Schurz et al., 2010; Wimmer et al., 2010). Hence, this region plays an important role in fast word recognition, as well as contributing to sub-lexical decoding by packaging together abstract visual features into corresponding letters and word forms (Cohen & Dehaene, 2004; Dehaene & Cohen, 2011; Price & Devlin, 2011b; Richlan, 2012).

A failure of this area to function normally is often viewed as the neural signature of some reading disorders (Dejerine, 1891). For instance, pure alexia is typically acquired following a lesion affecting the VWFA and is characterised by an inability to read written words efficiently, with some patients unable to read words at all. Patients who can read words do so abnormally slowly and employ an effortful letter-by-letter decoding of words resulting in word length effects, indicative of the struggle to employ a parallel whole-word and fluid reading strategy. The role of the VWFA beyond reading has been demonstrated by evidence that these patients may also present with impairments (abnormal response times) for stimuli that are as visually demanding as letters/words including abstract visual patterns, objects, and faces (e.g. Behrmann & Plaut, 2014; Roberts et al., 2013, 2015). Interestingly, word length effects and impairments for non-orthographic visual stimuli (including objects and faces) have also been noted in both children and adults with DD (Gabay et al., 2017a; Provazza et al., 2019b; Sigurdardottir et al., 2018), strengthening the account of a VWFA dysfunction. It is therefore plausible to hypothesise that a failure in its engagement may result in a visual processing deficit (in addition to phonology) in DD.

To investigate this, a recent study administered a battery of non-reading tasks to DD readers (Provazza et al., 2019b), whose efficacy in measuring visual processing deficits was demonstrated by an earlier study (Roberts et al., 2013). Specifically, two forced choice visual discrimination tasks using unfamiliar checkerboard and kanji stimuli (novel visual patterns) that do not map onto or require access to visual-phonological codes known to influence DD performance (Ramus & Szenkovits, 2008; Ziegler et al., 2010) were used. The design also allows the differentiation of accuracy and speed to evaluate the critical features of any processing deficit observed (such as that underlined by Wimmer, 1993). Furthermore, using unfamiliar and novel stimuli avoids underestimating the severity of the visual impairment by excluding the possible contribution of top-down facilitatory processes for familiar stimuli (e.g. semantic representations, visual-phonological mapping) and thus provides a finer quantification of visual processing per se. The stimuli also varied in visual complexity, based on the theoretical position that a dysfunction in the VWFA would disproportionately affect visually demanding stimuli (Roberts et al., 2013). Indeed, this is supported by patients with acquired dyslexia who show abnormal response latencies and a performance decrease as visual complexity of the stimuli increased (Roberts et al., 2013), impairments quantified by the extent of reading deficiency (e.g. abnormally slow reading and word length effects).

The results of the above study (Provazza, Adams, et al., 2019) extended these findings to a DD population, who showed a pattern of performance analogous to pure alexic patients (Roberts et al., 2013), displaying dramatically extended response latencies when processing visual stimuli and discriminating between novel visual patterns. These findings illustrate that DD individuals present not only with phonological impairments but also difficulties in processing unfamiliar non-verbal visual materials, and when combined with previous reports, suggests that DD may be characterised by impaired phonological and visual mechanisms that underpin reading.

It is worth noting that the aforementioned study, as well as most previous studies demonstrating visual impairments in DD, was conducted in an English population. As far as we are aware, despite offering a crucial link between visual processing deficits and reading impairments, this has yet to be investigated in DD individuals who read orthographies of differing depth. It is well-established that the behavioural manifestation of DD varies across languages according to the depth of the writing system (Landerl et al., 1997; Provazza, Giofrè, et al., 2019; Richlan, 2014; Wimmer, 1993) and one may speculate that some differences in visual processing may be found among these orthographies as well. Early evidence of cross-linguistic differences was provided by studies using rapid automatised naming (RAN) (Norton & Wolf, 2012; Wolf et al., 1994). Notwithstanding current debate about the nature of the cognitive underpinnings of the RAN-reading relationship (see, e.g., Georgiou & Parrila, 2020), one hypothesis suggests poor readers perform worse in RAN tasks because of an underlying visual processing impairment (Stainthorp et al., 2010). Intriguingly, although RAN seems to predict poor reading across a range of orthographies (e.g. Landerl et al., 2013), it appears to have more importance in shallow than in deep orthographies (Helland & Morken, 2016; Torppa et al., 2013; Wolf & Bowers, 1999).

There is now a body of evidence suggesting an impairment in the rapid processing of complex, unfamiliar visual stimuli may be a contributing factor in the reading and associated visual deficits observed in DD individuals. By adopting a cross-linguistic approach, the novelty and aim of the current study was to investigate the extent to which deficits in visual processing skills manifest differentially in DD readers of shallow (Italian) and deep (English) orthographies by comparing their performance on two visual discrimination tasks (Roberts et al., 2013) with that of two groups of typically developing readers (TDR). As described above, the sensitivity of these tasks at capturing visual processing deficits has been validated in patients with acquired (Roberts et al., 2013) and developmental (Provazza, Adams, et al., 2019) dyslexias. Additionally, we administered the digit span task (WAIS-IV; Wechsler, 2008), which measures verbal short-term memory and working memory, two of the most consistent associated deficits observed in DD (Jeffries & Everatt, 2004; Menghini et al., 2011; Trecy et al., 2013; Wang & Gathercole, 2013), which seem to be caused by a deficiency in or access to phonological representations (Ramus & Szenkovits, 2008).

Based on the evidence reviewed above, it was predicted that individuals with DD will perform more poorly than typically developing readers on the visual processing tasks, with DD readers of the shallow orthography exhibiting a more severe impairment particularly in speed of processing. Moreover, individuals with DD will also perform more poorly than typically developing readers in the phonological task as stipulated by the phonological deficit hypothesis.

## Method

### Participants

Thirty-six university students with DD participated. Italian speakers (*N* = 18) were recruited at the University of Calabria (5 males; age range 19–26; *M*_years_ = 21; SD = 2.35), and British English speakers (*N* = 18) were recruited at Liverpool John Moores University (5 males; age range 19–27; *M*_years_ = 21.8; SD = 2.29). All participants were in receipt of a formal diagnosis of dyslexia (supplied by a registered assessor of SpLD). Each DD group has been contrasted to a group of TDR matched for age, language, and gender. This included 18 Italian speakers recruited at the University of Padova (6 males; age range 19–25; *M*_years_ = 21.17; SD = 1.86) and 18 British English speakers recruited at Liverpool John Moores University (7 males; age range 19–28; *M*_years_ = 21.8; SD = 2). All groups did not differ for gender, *χ*^2^(3) = 0.70, *p* = 0.873, Cramer’s *V* = 0.099, or age, *F*(3,68) = 0.509, *p* = 0.677, *η*^2^_p_ = 0.022. The TDR did not have language disorders or ADHD.

The reading level of English DD and TDR groups was assessed using two reading tasks (i.e. word and non-word reading, Roberts et al., 2010). As expected, the TDR group outperformed the DD group in both word (*U* = 233.5, *n* = 36 *p* = 0.02) and non-word reading (*U* = 258.5, *n* = 36 *p* = 0.002). The reading level of the Italian DD and TDR groups was also assessed using two reading tasks (word and non-word reading, Cornoldi & Montesano, 2020). Both accuracy and speed were evaluated in this group. As expected, the TDR group outperformed the DD group in both fluency (word reading *t*(34) = 5.97, *p* < 0.001) (non-word reading *t*(34) = 6.34, *p* < 0.001) and accuracy (word reading *U* = 312, *n* = 36 *p* < 0.001) (non-word reading *U* = 242.5, *n* = 36 *p* = 0.01). Medians, interquartile ranges for the error rates of the two groups, and group comparisons in terms of odds ratio are displayed in Table 1. Fluency measures for the Italian group are reported in Table 2.

**Table 1:** Median error rates (interquartile range) and odds ratio (OR) for Italian and English developmental dyslexics (DD) and typically developing readers (TDR) in the reading tasks

	Italian			English		
	DD	TDR	OR	DD	TDR	OR
Word errors	6.50 (6.75)	1.00 (2.00)	17.24	6.00 (6.50)	2.00 (4.00)	4.26
Non-word errors	5.50 (3.50)	3.00 (3.25)	6.02	4.00 (8.25)	2.00 (2.00)	5.70

**Table 2:** Mean (standard deviation) and Cohen’s *d* for word and non-word fluency for Italian developmental dyslexics (DD) and typically developing readers (TDR)

	Italian		
	DD	TDR	*d*
Word errors	2.70 (.87)	4.51 (.90)	.88
Non-word errors	1.85 (.45)	2.88 (.51)	2.92

The study was approved by the RES Committee North West Liverpool Central (15/NW/0461), and by the ethics committees of the University of Calabria and the University of Padova, and written consent was obtained from all participants. The participants did not have sensory disorders (i.e. visual problems).

### Materials

#### ***Visual processing tasks (***Roberts et al., 2013***)***

Two visual discrimination tasks were administered to assess visual abilities and are described below. For each of these tasks, RT and accuracy data were collected.

#### Checkerboards

**Fig. 1:**
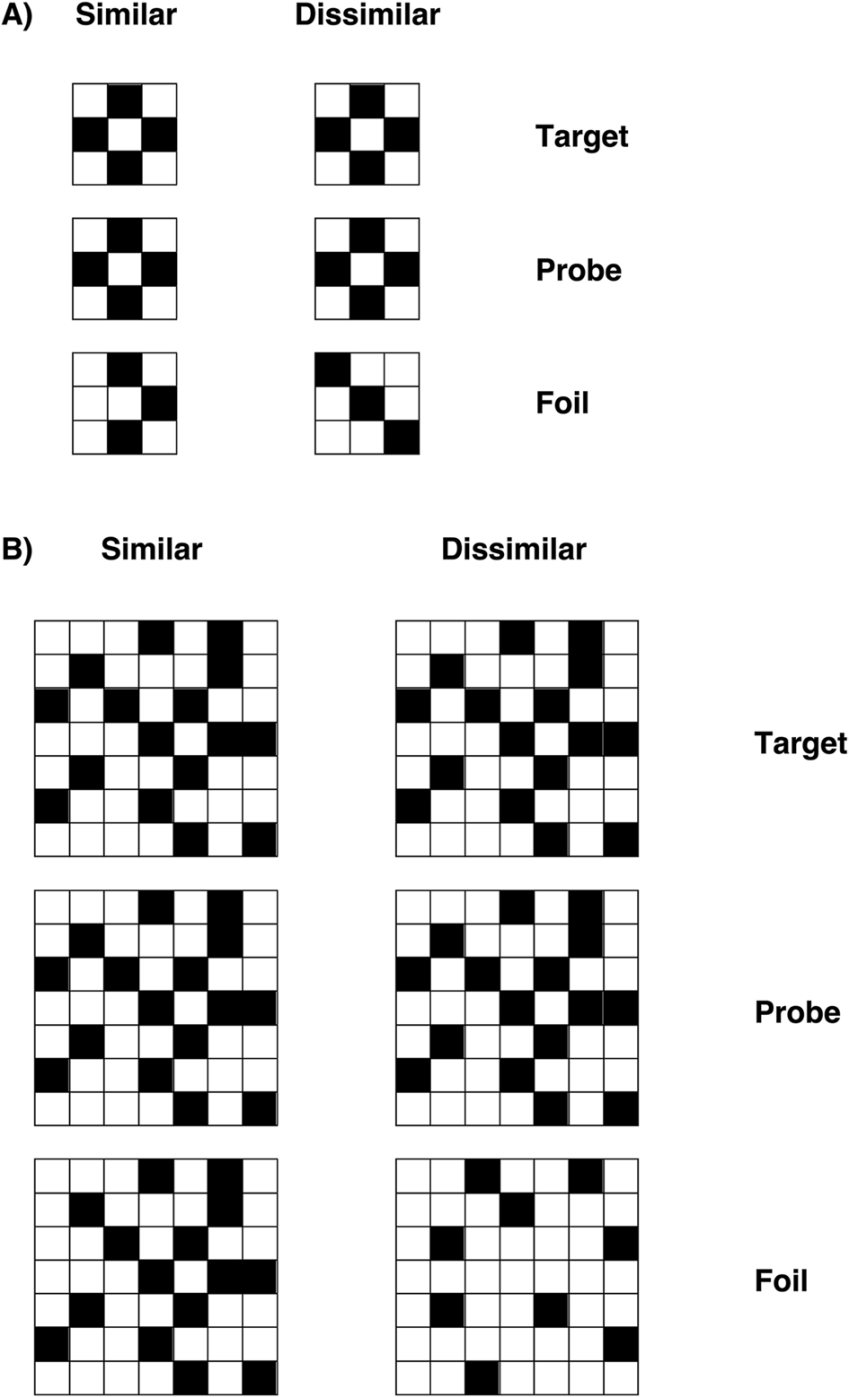
Example checkerboard stimuli for **A** visually simple condition and **B** visually complex condition with similar and dissimilar foils (Roberts et al., 2013)

A set of 32 target black and white checkerboards were used (Fig. 1). The number of squares in each matrix was either 9 (3 × 3) or 49 (7 × 7), forming the visually simple (*N* = 16) and visually complex (*N* = 16) sets, respectively. Grids were constructed by avoiding placement of blocks of the same colour together or any other regularity in the patterns (that might simplify visual processing). Stimuli were used to form a triad-based matching-to-sample task, in which the probe was flanked either above or below by the target and foil. The position (above/below) of target and foil was randomised. Two types of foil (total of *N* = 32) were created and paired with each target checkerboard: the similar condition (*N* = 16) reflected foil patterns that differed by only one block from the target pattern; the dissimilar (*N* = 16) condition reflected foils that differed from the target considerably (by several blocks), such that each foil could be easily distinguished (a total of four conditions: simple target similar foil *N* = 16; simple target dissimilar foil *N* = 16; complex target similar foil *N* = 16; complex target dissimilar foil *N* = 16). Three vertically aligned checkerboards appeared on the screen for each trial, presented randomly across conditions. The central checkerboard was the probe stimulus, and the participants had to decide whether the top or bottom checkerboard matched the central one (i.e. they had to identify the target), by pressing two different keys on the keyboard (“N” for the stimulus below and “Y” for the stimulus above).

Participants completed the experiment in a laboratory cubicle with no windows, and one ceiling light. Stimulus presentation was controlled using E-prime software (Schneider, Eschman, & Zuccolotto, 2002) on a 17″ LCD panel display at 1024 × 768 resolution and ~ 60-Hz refresh rate. Participants were seated approximately 50 cm from the screen and were asked to respond as quickly and accurately as possible. Stimuli remained on screen until a response was given. The next trial began after a 1-s pause.

#### Kanji

**Fig. 2:**
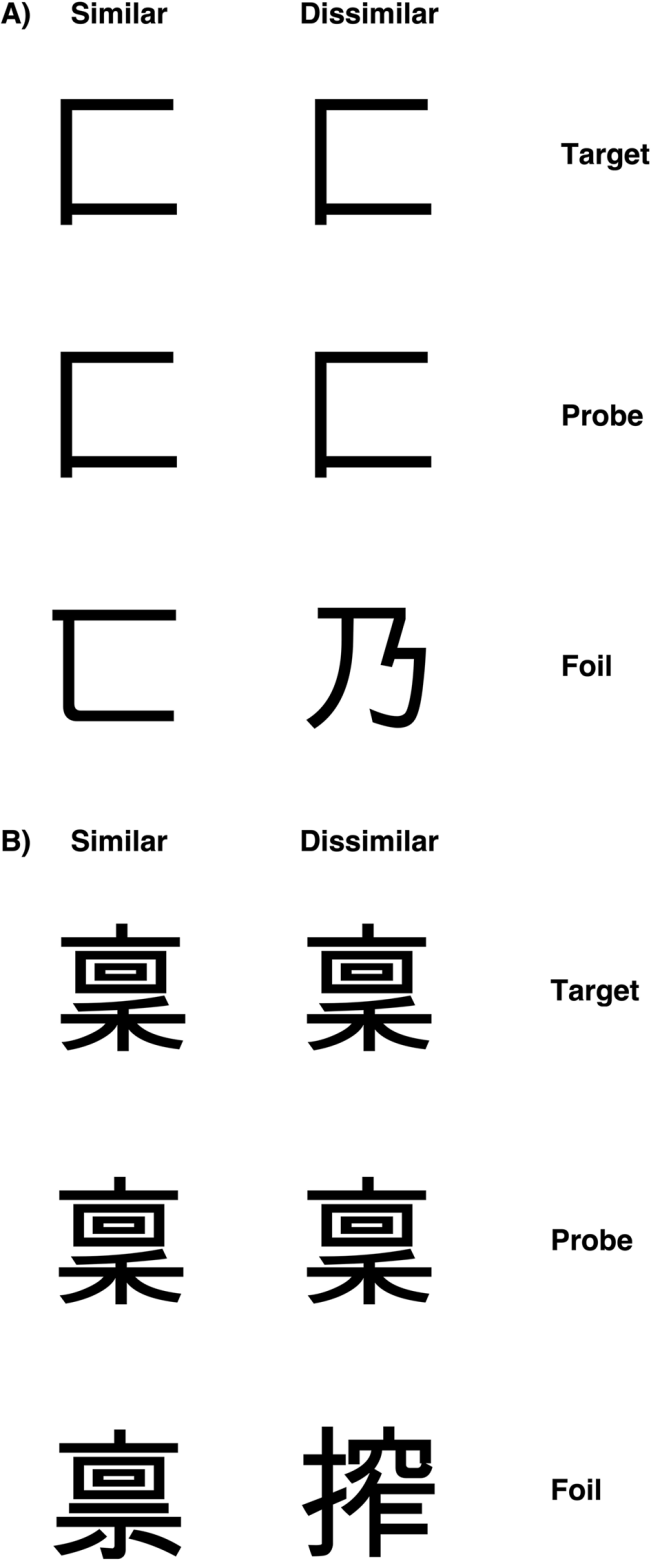
Example kanji stimuli for **A** visually simple condition and **B** visually complex condition with similar and dissimilar foils (Roberts et al., 2013)

A set of 60 single kanji characters were used (Fig. 2). Visual complexity was defined in terms of the number of strokes in each character. Characters with 2–4 strokes constituted the simple items (*N* = 30), and those with 13 strokes formed the complex set (*N* = 30). Again, each target character appeared in a matching-to-sample triad. The probe was placed in the centre with the target and foil above or below. The position of the target was randomised across trials. In half the trials, the foil was a character differing only slightly from the target to give the similar condition; in the other half, a character differing from the target considerably was selected for the dissimilar condition (a total of four conditions: simple target similar foil *N* = 15; simple target dissimilar foil *N* = 15; complex target similar foil *N* = 15; complex target dissimilar foil *N* = 15). Three vertically aligned kanji appeared on the screen for each trial, presented randomly across each condition. The central kanji was the probe stimulus, and the participants had to decide whether the top or bottom kanji matched the central one, by pressing two different keys on the keyboard (“N” for the stimulus below and “Y” for the stimulus above). The procedure was identical to that described for checkerboard tasks.

#### ***Phonological tasks (WAIS-IV, ***Wechsler, 2008***)***

To investigate phonological processing, the digit span task was administered following instructions in the WAIS-IV administrator manual. This consists of three subtasks: digit forward, in which participants were instructed to recall as many of the digits as possible in the same order they were presented; digit backward, in which participants had to recall the digits in the reverse order; and digit sequential, which required participants to recall the digits in ascending order of magnitude. The digit span test score is obtained by summing scores across the three subtasks (Wechsler, 2008).

### Statistical analyses

#### Visual processing tasks

Analyses of the visual discrimination tasks were performed using generalised linear mixed models (GLMM Pinheiro & Bates, 2000) using the “lm4” package (Bates et al., 2015). GLMM is a robust analysis that allows controlling for the variability of items and subjects, limiting the loss of information due to the prior averaging of the by-item and by-subject analyses (Baayen et al., 2002).

To obtain the *p*-values for the random effects, a null model with both random effects was compared with a model in which only one random effect was included. *P*-values for fixed effects were obtained using the package “car” with the type II Wald chi-square tests (Fox & Weisberg, 2019). Figures were obtained using the package “ggplot2” (Wickham, 2016).

In each model, participant and trial were identified as random variables, whilst group (TDR and DD), language (English and Italian), complexity (complex and simple), and similarity (similar and dissimilar) were included as fixed effects. The function “lmer” was used to perform the analyses concerning reaction times (RT), whilst “glmer” was used to fit the analyses on accuracy. An optimiser was used for the analyses performed fitted with “glmer”, i.e. “bobyqa”.

As far as the accuracy is concerned, generalised linear mixed models do not rely on a normal distribution but are generally fitted using a binomial distribution. Binomial distribution is generally used when the response variable (sometimes referred as dependent variable) is binomial in nature, as in this case 0 incorrect, whilst 1 correct. This approach is statistically superior for several reasons, including the use of all the available information, and the fact that it does not assume normality. In fact, ANOVA is inappropriate with a categorical response variable and leads to invalid results (see Jaeger, 2008, for an extensive discussion of the issue).

Producing effect size measures for generalised linear mixed models remains challenging. One measure, which is often advocated by the current literature, is the intraclass correlation (ICC; Bolker et al., 2009). In the current report, adjusted (ICC_Adj_) or conditional (ICC_Cond_) ICCs have been used and are reported alongside the fit of the model (see Hox, Moerbeek, & Van de Schoot, 2017, for a thorough discussion of the issue).

## Results

Median, interquartile ranges, and odds ratio for RT and accuracy of the two groups in the visual discrimination tasks and in the digit span task are displayed in Table 3.

**Table 3:** Median (interquartile range) and odds ratio (OR) for Italian and English developmental dyslexics (DD) and typically developing readers (TDR) in visual and phonological tasks

	**Italian**			**English**		
	**DD**	**TDR**	**OR**	**DD**	**TDR**	**OR**
**Visual discrimination**						
Checkerboard RT						
Complex dissimilar	2515 (7918.12)	1427 (2307.85)	10.19	1951 (1833.25)	1339 (1505.06)	5.40
Complex similar	9042 (17,645.87)	4268 (4966.36)	17.56	6407 (10,363.38)	5492 (6371.96)	3.08
Simple dissimilar	2212 (2701.81)	1013 (1203.09)	50.29	1422 (1552.77)	1169 (798.50)	5.91
Simple similar	2360 (5574.96)	1213 (1379.88)	17.56	1722 (1751.04)	1390 (1022.67)	4.75
Kanji RT						
Complex dissimilar	2253 (3656.07)	1245 (926.57)	4.93	1575 (1817.33)	1261 (692.25)	5.21
Complex similar	3234 (6017.92)	1695 (2012.74)	10.01	2422 (3092.57)	1808 (2315.54)	5.60
Simple dissimilar	1728 (2781.50)	966 (652.48)	3.96	1142 (1049.47)	916 (692.25)	3.69
Simple similar	2640 (3678.37)	1366 (1095.32)	3.69	1651 (2742.24)	1421 (2044.24)	5.40
Checkerboard accuracy						
Complex dissimilar	1.00 (0.06)	1.00 (0.12)	1.82	1.00 (0.12)	1.00 (0.06)	5.02
Complex similar	0.94 (0.31)	0.97 (0.31)	3.56	0.88 (0.50)	0.94 (0.37)	1.81
Simple dissimilar	1.00 (0.06)	1.00 (0.10)	1.00	1.00 (0.06)	1.00 (0.19)	1.00
Simple similar	1.00 (0.12)	0.94 (0.25)	3.13	0.97 (0.25)	0.97 (0.19)	1.34
Kanji accuracy						
Complex dissimilar	1.00 (0.07)	1.00 (0.17)	3.13	1.00 (0.10)	1.00 (0.10)	1.00
Complex similar	1.00 (0.24)	0.97 (0.31)	2.76	0.93 (0.38)	0.97 (0.10)	4.34
Simple dissimilar	1.00 (0.07)	1.00 (0.10)	1.00	1.00 (0.07)	1.00 (0.10)	1.00
Simple similar	0.97 (0.17)	0.98 (0.27)	1.29	0.97 (0.13)	0.93 (0.13)	1.43
**Phonology**						
Digit span	21.00 (15)	31.50 (21)	39.72	24.00 (16)	30.00 (14)	20.67

### Correlations

Spearman rank correlations were performed to evaluate the relationships between the tasks. The correlation matrix is displayed in Table 4. Moderated negative correlations were found between the RT of the visual discrimination tasks and the digit span forward _(_*r*_s_ ≤  − 0.454, *N* = 72, *p*s < 0.01), digit backward _(_*r*_s_ ≤  − 0.390, *N* = 72, *p*s < 0.01) and sequencing _(_*r*_s_ ≤  − 0.441, *N* = 72, *p*s < 0.01) tasks.

**Table 4:** Task correlations

	1	2	3	4	5	6	7
1 Checkerboard RT	-						
2 Checkerboard accuracy	.368**	-					
3 Kanji RT	.848**	298*	-				
4 Kanji Accuracy	.222	.642**	.269*	-			
5 Digit forward	-.364**	.018	-.454**	.057	-		
6 Digit backward	-.366**	.090	-.390**	.081	.608**	-	
7 Digit sequencing	-.351**	.086	-.441**	.116	.543**	.507**	-

**p < .01; *p < .05

### Visual processing tasks

#### Reaction times

##### Checkerboard

Random effects were statistically significant (*p* < 0.001). All fixed effects referred to the checkerboard task are presented in Table 5 (model 1 = checkerboard RT). The main effects were all statistically significant (*p*s < 0.026). The two-way interactions were statistically significant (*p*s < 0.004) except for the language × similarity (*p* = 0.563).

**Table 5:** Fixed effects

	Model 1		Model 2		Model 3		Model 4	
	*χ*^2^(1)	*p*	*χ*^2^(1)	*p*	*χ*^2^(1)	*p*	*χ*^2^(1)	*p*
Group	**31.35**	**.000**	**39.01**	**.000**	0.66	.418	0.21	.645
Language	**4.98**	**.026**	**8.16**	**.004**	0.11	.741	1.88	.170
Complexity	**270.05**	**.000**	**27.13**	**.000**	**16.39**	**.000**	3.04	.081
Similarity	**228.09**	**.000**	**64.53**	**.000**	**37.81**	**.000**	**44.50**	**.000**
Group * language	**12.48**	**.000**	**12.98**	**.000**	**9.06**	**.003**	3.80	.051
Group * complexity	**82.71**	**.000**	**23.72**	**.000**	0.64	.423	0.68	.410
Language * complexity	**8.21**	**.004**	2.04	.153	2.24	.134	0.20	.655
Group * similarity	**56.55**	**.000**	**78.50**	**.000**	1.39	.238	2.29	.131
Language * similarity	0.33	.563	**5.21**	**.022**	1.20	.274	0.51	.476
Complexity * similarity	**173.02**	**.000**	0.36	.549	**3.84**	**.050**	0.33	.568
Group * language * complexity	**36.95**	**.000**	3.46	.063	0.04	.842	**8.51**	**.004**
Group * language * similarity	**23.85**	**.000**	**27.62**	**.000**	0.29	.587	0.06	.806
Group * complexity * similarity	**38.15**	**.000**	0.03	.873	0.63	.429	1.91	.167
Language * complexity * similarity	0.07	.788	0.02	.887	0.01	.927	0.04	.845
Group * language * complexity * similarity	**14.38**	**.000**	0.89	.345	1.10	.295	0.06	.802

Model 1 = checkerboard RT; Model 2 = kanji RT; Model 3 = checkerboard accuracy; Model 4 = kanji accuracy

**Bold** denotes statistical significance

**Fig. 3:**
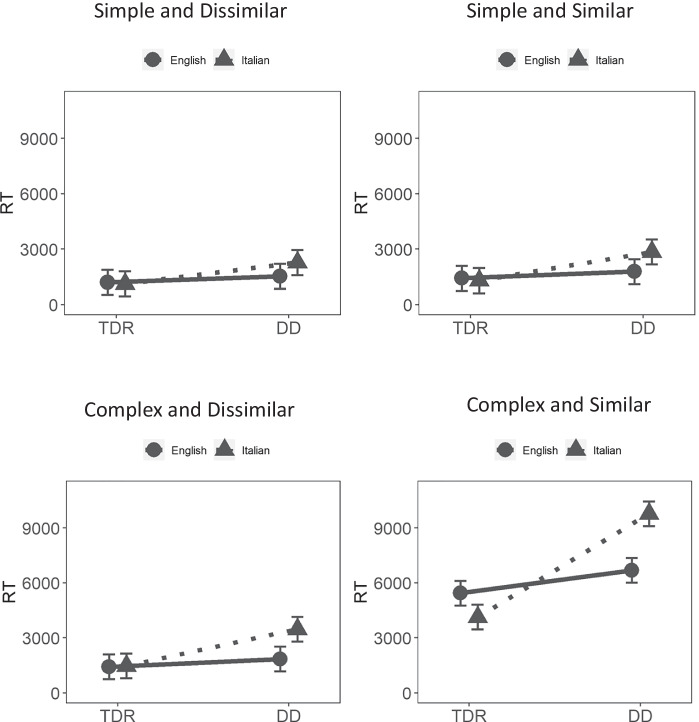
Checkerboard results. TDR, typically developing readers; DD, developmental dyslexics; RT, reaction times

The four-way interaction between group, language, visual complexity, and visual similarity was statistically significant, *χ*^2^(1) = 14.38, *p* < 0.001, ICC_Adj_ = 0.175, ICC_Cond_ = 0.109. Figure 3 shows that participants in the TDR group in the two languages performed similarly, outperforming participants with DD, who in general presented with slower RT. Intriguingly, some differences emerged in the complex similar condition, which was the most visually challenging condition. In this condition, RTs were generally higher and DD Italian participants performed poorly compared with all the other groups, including DD English participants.

##### Kanji

Random effects were statistically significant (*p* < 0.001). All fixed effects referred to the kanji task are presented in Table 5, ICC_Adj_ = 0.257, ICC_Cond_ = 0.208 (model 2 = kanji RT). The main effects were all statistically significant (*p*s < 0.004). The two-way interactions were statistically significant (*p*s < 0.022) except for language × complexity (*p* = 0.153) and complexity × similarity (*p* = 0.549).

**Fig. 4:**
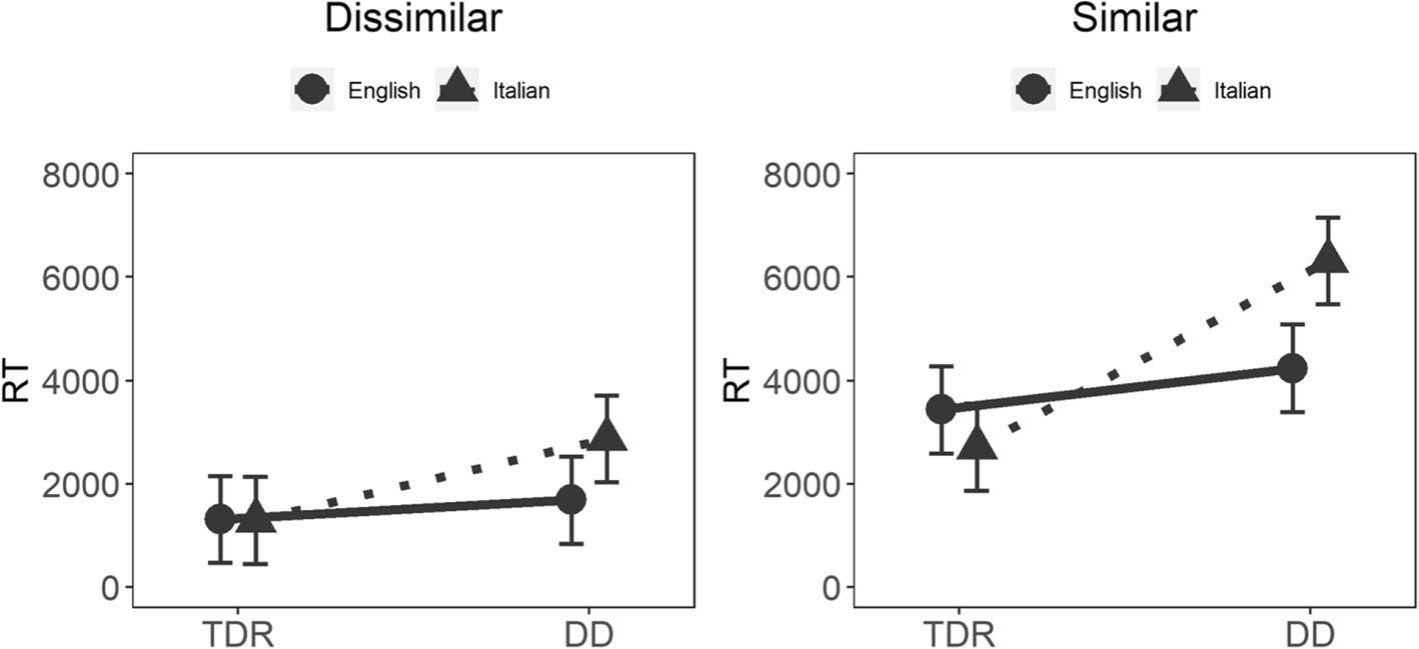
Kanji results*.* TDR, typically developing readers; DD, developmental dyslexics; RT, reaction times

The four-way interaction and three out of four of the three-way interactions were not statistically significant. However, the interaction between group, language, and visual similarity was statistically significant, *χ*^2^(1) = 27.62, *p* < 0.001. Figure 4 shows that participants in the TDR group in the two languages performed similarly, outperforming participants with DD, who in general presented with slower RTs. Intriguingly, some differences emerged in the visually challenging similar condition. In this condition, RTs were generally higher and DD Italian participants performed quite poorly as compared with all the other groups, including DD English participants.

#### Accuracy

##### Checkerboard

Random effects were statistically significant (*p* < 0.001). All fixed effects on accuracy in the checkerboard task are presented in Table 5 (model 3 = checkerboard accuracy), ICC_Adj_ = 0.284, ICC_Cond_ = 0.211. Only complexity and similarity were statistically significant (*p*s < 0.001).

**Fig. 5:**
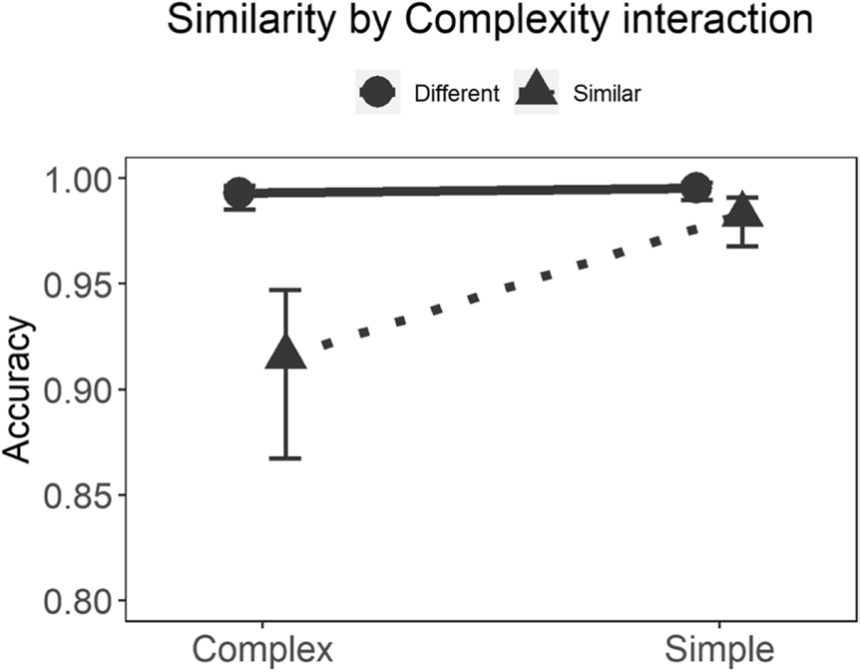
Checkerboard two-way (similarity × complexity) interaction on accuracy

The four-way and all the three-way interactions were not statistically significant. As for the two-way interactions, only the interaction between complexity and similarity was statistically significant (Table 5). Figure 5 shows that the complex similar condition was the most difficult, with higher error rates. This effect was evident across all the participants, with no distinction between DD and TDR. Nonetheless, performance in all groups was still high and participants made very few errors.

##### Kanji

Random effects were statistically significant (*p* < 0.001). All fixed effects are presented in Table 5 (model 4 = kanji accuracy), ICC_Adj_ = 0.345, ICC_Cond_ = 0.298.

**Fig. 6:**
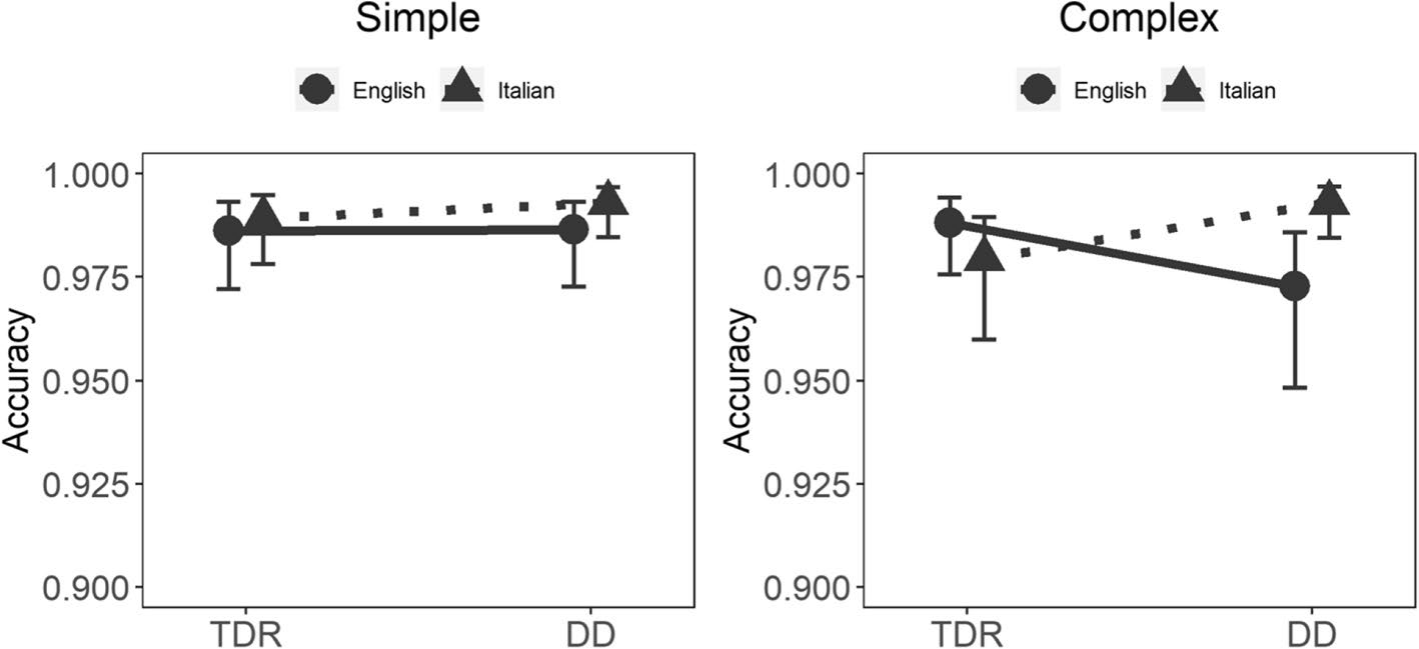
Kanji results. TDR, = typically developing readers; DD, developmental dyslexics

All four-way interactions were not statistically significant. As for the three-way interactions, only the interaction between group, language, and complexity was statistically significant (Table 4). Figure 6 shows that in the complex condition DD English participants had somewhat lower accuracy, 0.973, with 95% CIs (0.948, 0.985); however, inspection of the confidence intervals showed that this performance is highly overlapping with the performance of English TDRs, 0.988, with 95% CIs (0.976, 0.994). Taken together, these results indicate that the overall performance was extremely high and that participants made very few errors.

### Phonological processing

ANOVA was conducted to compare the performances of the four groups. The results showed that the DD group performed worse than TDR group, *F*(1,68) = 62.46, *p* < 0.001, *η*^2^_p_ = 0.480, with a large effect size regardless of the language *F*(1,68) = 3.03, *p* < 0.086, *η*^2^_p_ = 0.043. The interaction group × language was not statistically significant *F*(1,68) = 1.41, *p* = 0.240, *η*^2^_p_ = 0.020 with a small effect size, indicating that there was no difference in terms of phonological processing between the DD groups in the two languages.

## Discussion

The aim of this cross-linguistic investigation was twofold. First, and most importantly, to explore the extent to which non-reading visual deficits are observed in DD readers of shallow (Italian) and deep (English) orthographies. Second, to evaluate the presence of a phonological impairment across these orthographies, in accordance with the phonological deficit hypothesis of DD. The overarching objective was to investigate the hypothesis that reading and associated non-reading deficits observed in DD are anchored, albeit differentially, by impairments to visual and phonological mechanisms that underpin reading.

As predicted, all DD participants presented with a deficit in processing visual stimuli, aligning with previous findings (Gabay et al., 2017b; Giofrè et al., 2019a; Jozranjbar et al., 2020; Provazza et al., 2019b; Sigurdardottir et al., 2018; Sigurdardottir et al., 2015; Vogel et al., 2014). This was evident in RT, with no difference in accuracy. Although the importance of accuracy is not questioned, our results underline the significance of considering both accuracy and speed of processing, the latter being more sensitive at capturing visual impairments in developmental (Provazza et al., 2019b) as well as in acquired (Roberts et al., 2013) reading disorders. The pattern of impairment was comparable to that shown in acquired pure alexic patients (Roberts et al., 2013), whose reading and non-reading visual deficits are attributable to VWFA lesions (Starrfelt & Shallice, 2014). Since the role of the VWFA is not exclusive to letter strings but extends to other visually demanding stimuli (Price & Devlin, 2003, 2011a), a dysfunction of this region may account for our results.

As well as DD being heterogeneous in terms of how impaired phonological and visual mechanisms might contribute to its manifestation, our results demonstrate this heterogeneousness is amplified as a function of orthographic depth. The Italian DD group performed significantly worse than the English DD and TDR groups in the visual tasks, and these differences were particularly evident in the visually complex and similar conditions. These behavioural results might be explained by a common hypoactivation of the VWFA in DD (Martin et al., 2016; Richlan, 2020), the degree of which varies according to the orthographic depth of the writing system (Italian—shallow; English—deep). Thus, experience and mastery of a specific writing system may shape the emerging functionality, distribution, and pattern of activity in the occipito-temporal cortex during reading, whilst also accommodating stimuli that, by their very nature, necessitate similar processing to letters. Indeed, studies have demonstrated that literacy acquisition improves visual processing and reorganises the occipito-temporal cortex (Behrmann & Plaut, 2020; Dehaene et al., 2015).

Behaviourally, it would be reasonable to postulate that individuals with DD are impaired in processing visual information, and this should be more evident in shallow than deep orthographies based on, for example, the emerging functionality and reliance on visual occipito-temporal areas when learning to read. This is supported by our results, and findings of previous studies demonstrating an interconnectedness of visual-phonological impairment in DD, with visual processing being more crucial in readers of shallower orthographies (Helland & Morken, 2016; Wimmer et al., 2000) and phonological factors being more important in readers of deeper orthographies (Elliot & Grigorenko, 2014; Georgiou et al., 2008; Share, 2008).

According to the psycholinguistic grain size theory (Ziegler & Goswami, 2005), readers of shallow orthographies rely on smaller grain sizes when reading (although a whole-word reading strategy is still employed by readers of shallow orthographies, see Marinelli et al., 2016, for further details), whereas readers of deep orthographies rely on larger grain sizes, which may be more reliable in an inconsistent writing system. As we pointed out above, the VWFA is engaged in a whole-word recognition strategy but also shows increased activation in response to sub-lexical decoding (Richlan, 2014; Wimmer et al., 2010). One could speculate, therefore, that the behavioural observation of slow reading in shallow orthographies may be due to an inefficient lexical and sub-lexical process attributable to consistent hypoactivation of the VWFA (Richlan et al., 2010; Wimmer et al., 2010). There is much to be learned about the putative dysfunction of the VWFA and how this differs in DD readers of different orthographies, and this would be an interesting line of enquiry for future research.

What we do know from the existing literature is that the VWFA shows greater activation in response to unfamiliar letter strings compared to familiar letter strings (Price & Devlin, 2011a; Schurz et al., 2010). In deep orthographies such as English, a greater reliance upon orthographic and phonological knowledge through the employment of larger units (whole words) is a prerequisite for mastering reading due to the nature of the orthography, and hence, the early acquisition of a parallel reading strategy (larger grain size employment) may be essential. Indeed, readers of deep orthographies show stronger influences from whole-word phonology even when reading novel letter strings (Provazza et al., 2019a; Ziegler & Goswami, 2005). Conversely, a whole-word recognition strategy is less important in shallow orthographies such as Italian, due to the consistency of the speech to sound mapping (see, e.g., Marinelli et al., 2016). These orthographic differences may result in a reduced reliance on the VWFA in English compared to shallow orthographies such as Italian. As such, a more marked hypoactivation of the VWFA in shallow orthographies could better account for reading difficulties. Interestingly, with respect to DD in deep orthographies, meta-analytic findings showed a higher convergence of hypoactivation was found in the inferior frontal gyrus, pars triangularis. This area is implicated in phonological and semantic reading processes (Price, 2012) and might explain poor reading in DD in deep orthographies, in terms of lexico-semantic and phonological reading difficulties (Martin et al., 2016).

Turning to phonology, our results also demonstrated that all DD participants, irrespective of orthography, were less accurate on phonological tasks (with large effect sizes), compared to the TDR groups. These findings were expected on the basis of previous studies on impaired short-term memory in DD and offer support for the phonological deficit hypothesis (Ackerman & Dykman, 1993; Goswami, 2002; Ramus & Szenkovits, 2008; Snowling, 1995; Snowling & Hulme, 1994). Furthermore, it is worth noting that the measures of phonological memory (either short-term memory or working memory) were negatively correlated with both the checkerboard and kanji RT.

Our study has employed only behavioural measures to evaluate the performance of DD participants and much research remains to be done to extend these findings. One line of enquiry would be to confirm our behavioural evidence using neuroimaging, for instance, functional magnetic resonance imaging (fMRI), and methods including total brain volume, voxel- and surface- based morphometry, white matter, diffusion imaging, brain gyrification, and tissue metabolite to evaluate the hypoactivation of the VWFA in DD (see, e.g., Adrián-Ventura et al., 2020; Paulesu et al., 2001; Ramus et al., 2018; Richlan, 2014). Using fMRI to compare volume, connectivity, and patterns of activation in occipito-temporal cortex of DD readers of different orthographies would be a valuable tool to test our hypothesis of visual impairment in DD.

The sample of our study only allowed us to test Italian and English participants and it would be useful to conduct larger studies across a variety of orthographies, and specifically, to investigate our prediction that DD manifests from and encompasses underlying problems in both the phonological and visual domains. We hypothesise that impairments in visual and phonological processing may reflect a continuum, and therefore, individuals should present with either a deficit in the visual or in the phonological domain or with a combination of both to some extent dependent on orthographic depth. Furthermore, our participants were highly educated adults, likely to be motivated in their desire to read, and it is conceivable that they may have adopted compensatory strategies (Warmington et al., 2013). It would therefore be useful to explore visual and phonological impairments in different demographic groups over time.

One approach would be to conduct longitudinal studies to investigate the impact of visual and phonological processing skills on the trajectory of reading development across a range of orthographies. Large-scale longitudinal studies would help clarify whether visual processing deficits play a causal role in poor reading. Establishing a link between visual processing deficits and DD(e.g. measuring the extent that visual processing prior to the establishment of reading predicts later reading attainment; see Rauschenberger, Baeza-Yates, & Rello, 2020) as well as demonstrating that training visual processing skills may lead to improvement in reading ability would strengthen our hypothesis of a fundamental role of visual processing in DD. Furthermore, longitudinal cross-linguistic comparisons would help corroborate the differential role of visual processing across orthographies of varying depth. Life-long experience of reading a particular orthography appears to affect activation patterns in VWFA (Stanislas Dehaene et al., 2015) and comparable visual processing skills may have differential impact related to the orthographic depth of the writing system.

Finally, our study presents with some limitations. Participants were not matched for IQ; however, we would expect differences in IQ to be insignificant in this sample of academically able adults in higher education and thus would not impact substantially the conclusions drawn. Indeed, we have suggested above that it would be useful to test different demographic groups of participants. IQ is a generic and broad concept, and the use of intelligence batteries in participants with DD and learning disabilities has been questioned with respect to biases in the use of intelligence estimates (Giofrè & Cornoldi, 2015; Giofrè et al., 2019a). Specifically, IQ differences might reflect artefacts of the battery in use, rather than differences in the proposed latent variables. We do acknowledge that perhaps in more differentiated samples, the use of intelligence tests may be meaningful (see, e.g., Kemp et al., 2009; Paizi et al., 2013). A further limitation is sample size. To address this, the analytic approach that we employed (i.e. generalised linear mixed models) strengthened the experimental power of the by-subject and by-item analyses and limited the loss of information due to the prior averaging of the by-subject and by-item analyses (Baayen et al., 2002; Paizi et al., 2013).

To summarise, our study aimed to investigate non-reading visual and phonological deficits in DD readers of shallow and deep orthographies, with the main objective of investigating the extent to which these deficits might contribute to the manifestation of DD across orthographic depth. Whilst the central diagnostic phenotype (reading) of DD is to some extent homogeneous and well-established across orthographic depth, the neurocognitive cause remains controversial (see Elliot & Grigorenko, 2014). Our results demonstrate that DD is not always the result of impaired phonological processing but may be better explained in terms of a dual phonology-visual processing impairment, both of which can be more or less critical, depending on the orthography.

Hence, the phonological deficit hypothesis may not always be a sufficient explanation of poor reading performance in DD and visual processing must be considered. DD individuals with impaired visual processing skills, leading them to struggle to interact effectively with a variety of visual information, whether letters, shapes, or objects, may encounter difficulties in learning to read. These adverse consequences may be heightened when reading a shallow orthography and result in an increased representation of this deficit in those identified as having DD. It is therefore important, especially for clinicians, to consider assessing visual and phonological abilities in DD, to fully capture the range of reading and non-reading difficulties individuals may present with, so that better remediation programmes can be developed (Rauschenberger et al., 2020).
